# Medical College of Buffalo

**Published:** 1853-05

**Authors:** 


*Medical College of Buffalo.—Annual Commencement.*—The commencement exercises of this institution took place on the 27th ultimo, at American Hall. The degree of Doctor in Medicine, was conferred upon eighteen candidates in course; and the honorary degree on Dr. Comfort Hamilton, of Ashtabula county, Ohio.

Prior to conferring the degrees, the President of the Council of the University, Hon. George W. Clinton, delivered an address replete with scholarship, and pronounced in the felicitous style which characterizes all the public efforts of this gentleman.

He was followed by Prof. Hamilton, to whom was assigned the customary charge to the graduates. This duty was performed by Prof. H. in an admirable manner.

Mr. Clinton's address, we are informed, will appear in one of the daily papers of this city.* We hope, also, that Dr. Hamilton's charge will be published. It will be read with interest, albeit medical addresses are so abundant, that, to borrow a mercantile phrase, the market is somewhat overstocked.

A number of the curators of the medical department of the University were present at the examination of the candidates for graduation, and expressed themselves highly gratified with the evidences of proficiency exhibited on that occasion.

The following is a list of the graduates:

Charles Ap Arthur Bowen, Hamilton, C. W.
Alfred Payson Crafts, Sodus, N. Y.
Joseph Rowe Smith, A. M., Buffalo, N. Y.
Albert Chaddock, Pavilion Centre, N. Y.
John Augustus Yeyte, Buffalo, N. Y.
Edwin Evans Kendall, Phillipston, Mass.
Thomas David Powell, Eden, N. Y.
Edwin Amsden, Gainsville, N. Y.
David Burnet McKay, Eddyville, N. Y.
George Edenmuller, Germany.
John Hart, Oswego, N. Y.

---

* It is contained in the Buffalo Daily Commercial Advertiser, April 28.

James Burley Rounds, Burford, C. W.
Owen Prouty Marsh, Springville, N. Y.
George E. Smith, Brighton, Michigan.
Albert Levant Cotes, Batavia, N. Y.
James Henry Moore, Davenport, N. Y.
Charles Lemon Dayton, Black Rock, N. Y.
Wm. Picket Bowers, Sinclairville, N. Y.

The medical faculty of the University, through the President of the Council, made public announcement, that, of the several graduates, Dr. Joseph R. Smith deserved especial distinction for a meritorious thesis, and a high order of attainment in the several branches of medical science.

